# Phenotypes of South Asian patients with atrial fibrillation and holistic integrated care management: cluster analysis of data from KERALA-AF Registry

**DOI:** 10.1016/j.lansea.2024.100507

**Published:** 2024-11-14

**Authors:** Yang Chen, Bi Huang, Peter Calvert, Yang Liu, Ying Gue, Dhiraj Gupta, Garry McDowell, Jinbert Lordson Azariah, Narayanan Namboodiri, Govindan Unni, Jayagopal Pathiyil Balagopalan, Gregory Yoke Hong Lip, Bahuleyan Charantharayil Gopalan, KERALA-AF Registry Investigators, Narayanan Namboodiri, A. Jabir, A. George Koshy, Geevar Zachariah, M. Shifas Babu, K. Venugopal, Eapen Punnose, K.U. Natarajan, Johny Joseph, C. Ashokan Nambiar, P.B. Jayagopal, P.P. Mohanan, Raju George, Govindan Unni, C.G. Sajeev, N. Syam, Anil Roby, Rachel Daniel, V.V. Krishnakumar, Anand M. Pillai, Stigi Joseph, G.K. Mini, Shaffi Fazaludeen Koya, Koshy Eapen, Raghu Ram, Cibu Mathew, Ali Faizal, Biju Issac, Sujay Renga, Jaideep Menon, D. Harikrishna, K. Suresh, Tiny Nair, S.S. Susanth, R.Anil Kumar, T.P. Abilash, P. Sreekala, E. Rajeev, Arun Raj, Ramdas Naik, S. Rajalekshmi, Anoop Gopinath, R. Binu, Jossy Chacko, P.T. Iqbal, N.M. Sudhir, Madhu Sreedharan, N. Balakrishnan, Muhammed Musthaffa, B. Jayakumar, Sheeba George, Anand Kumar, Thomas Mathew, V.K. Pramod, Muhammed Shaloob, Madhu Paulose Chandy, K.R. Vinod, Karuana Das, Z.Sajan Ahamad, Pramod Mathew

**Affiliations:** a Liverpool Centre for Cardiovascular Science at University of Liverpool, Liverpool John Moores University and Liverpool Heart and Chest Hospital, Liverpool, United Kingdom; b Department of Cardiovascular and Metabolic Medicine, Institute of Life Course and Medical Sciences, University of Liverpool, Liverpool, United Kingdom; c Department of Cardiology, The First Affiliated Hospital of Chongqing Medical University, Chongqing, People's Republic of China; d Department of Cardiovascular Medicine, The Second Affiliated Hospital, Jiangxi Medical College, Nanchang University, Nanchang, Jiangxi, People's Republic of China; e School of Pharmacy and Biomolecular Sciences, Liverpool John Moores University, Liverpool, United Kingdom; f Department of Clinical Research, Ananthapuri Hospitals and Research Institute, Thiruvananthapuram, India; g Department of Research, Global Institute of Public Health, Trivandrum, India; h Sree Chitra Tirunal Institute for Medical Sciences and Technology, Trivandrum, India; i Department of Cardiology, Jubilee Hridhayalaya, Jubilee Mission Medical College Hospital, Thrissur, India; j Department of Cardiology, Lakshmi Hospital, Kochi, India; k Danish Centre for Health Services Research, Department of Clinical Medicine, Aalborg University, Aalborg, DK-9220, Denmark; l Department of Cardiology, Ananthapuri Hospitals and Research Institute, Thiruvananthapuram, India

**Keywords:** Atrial fibrillation, Clustering analysis, Phenotype classification, ABC pathway, Kerala, South Asia

## Abstract

**Background:**

Patients with atrial fibrillation (AF) frequently experience multimorbidity. Cluster analysis, a machine learning method for classifying patients with similar phenotypes, has not yet been used in South Asian AF patients.

**Methods:**

The Kerala Atrial Fibrillation Registry is a prospective multicentre cohort study in Kerala, India, and the largest prospective AF registry in South Asia. Hierarchical clustering was used to identify different phenotypic clusters. Outcomes were all-cause mortality, major adverse cardiovascular events (MACE), and composite bleeding events within one-year follow-up.

**Findings:**

3348 patients were included (median age 65.0 [56.0–74.0] years; 48.8% male; median CHA_2_DS_2_-VASc 3.0 [2.0–4.0]). Five clusters were identified. Cluster 1: patients aged ≤65 years with rheumatic conditions; Cluster 2: patients aged >65 years with multi-comorbidities, suggestive of cardiovascular-kidney-metabolic syndrome; Cluster 3: patients aged ≤65 years with fewer comorbidities; Cluster 4: heart failure patients with multiple comorbidities; Cluster 5: male patients with lifestyle-related risk factors. Cluster 1, 2 and 4 had significantly higher MACE risk compared to Cluster 3 (Cluster 1: OR 1.36, 95% CI 1.08–1.71; Cluster 2: OR 1.79, 95% CI 1.42–2.25; Cluster 4: OR 1.76, 95% CI 1.31–2.36). The results for other outcomes were similar. Atrial fibrillation Better Care (ABC) pathway in the whole cohort was low (10.1%), especially in Cluster 4 (1.9%). Overall adherence to the ABC pathway was associated with reduced all-cause mortality (OR 0.26, 95% CI 0.15–0.46) and MACE (OR 0.45, 95% CI 0.31–0.46), similar trends were evident in different clusters.

**Interpretation:**

Cluster analysis identified distinct phenotypes with implications for outcomes. There was poor ABC pathway adherence overall, but adherence to such integrated care was associated with improved outcomes.

**Funding:**

Kerala Chapter of 10.13039/501100014741Cardiological Society of India.

**Research in context**

**Evidence before this study**

Prior to our investigation, there was a significant lack of data on multimorbidity in atrial fibrillation (AF) patients in India. The few existing AF cohort studies in India involved relatively small sample sizes and did not adequately capture the complex comorbid conditions prevalent in the Indian population. Additionally, AF in Indian and South Asian populations may present differently compared to other Asian regions, further underscoring the need for more comprehensive research.

**Added value of this study**

The significance of our study lies in its comprehensive contribution to the understanding of AF in India. Utilizing the largest known Indian prospective AF registry, the Kerala-AF registry, which involved the baseline assessment and 1-year follow-up of AF patients recruited from 53 medical centres across Kerala, India. We conducted a detailed cluster analysis of multimorbidity patterns within this unique population. This is the first study to identify distinct phenotypic clusters of AF patients in India, some of which were associated with adverse outcomes post-AF. Our findings offer valuable insights into the diverse clinical characteristics of AF patients in the region, potentially guiding more personalized treatment strategies.

Additionally, this study is the first to evaluate adherence to the Atrial Fibrillation Better Care (ABC) pathway in India, revealing varying levels of adherence across different clusters. We also found that adherence to the ABC pathway significantly improved outcomes, suggesting that widespread implementation of ABC management could play a critical role in reducing the AF burden in Indian and South Asian populations.

**Implications of all the available evidence**

Considering all available evidence and our findings, it becomes clear that the Kerala-AF registry offers valuable insights into the AF burden in India and South Asia. Our cluster analysis, identifying distinct multimorbidity patterns of AF in Indian and South Asian populations, provides a more nuanced understanding of the clinical characteristics associated with adverse outcomes post-AF. Additionally, our investigation into the ABC pathway adherence highlights the need for improved implementation of this management strategy, particularly in populations with high AF burden. The low adherence rates and their impact on outcomes emphasize the necessity for integrated care approaches in the region. Implementing the ABC management pathway more widely in India and South Asia could play a crucial role in reducing AF-related morbidity and mortality.

## Introduction

Atrial fibrillation (AF) is the most common arrhythmia seen in clinical practice and is associated with high mortality and morbidity from stroke, heart failure and dementia.^1^ Nonetheless, there are regional and ethnic differences in the epidemiology of AF and risk outcomes such as stroke and bleeding.^2^^,^^3^ Also, AF is not a homogeneous condition, and guidelines have suggested that AF is classified according to disease subtypes and pathological characteristics.^4^

AF is associated with frailty, multimorbidity, and polypharmacy, and some clinically complex domains may be identified.^5^ Also, different comorbidities tend to associate with each other; hence, simplified classifications by disease subtypes may be inaccurate in estimating the risk of adverse outcomes for AF patients.

Hierarchical clustering is an unsupervised machine learning approach that explores the multimorbidity pattern in a population, enabling samples to converge to form distinct clusters with high similarity based on pre-selected comorbidities and clinical features.^6^ One of the key strengths is that it can uncover hidden patterns and associations that might not be apparent through traditional analysis. This approach has been previously applied to AF patients from different regions, identifying different representative phenotypic clusters and demonstrating that these clusters were associated with different risks of adverse clinical outcomes.^7^^,^^8^ To date, no relevant studies have been performed in South Asia. Therefore, it is unclear what phenotypic clusters may exist in this population, how they are treated and what their outcomes are concerning contemporary guideline-recommended holistic or integrated care management.^9^^,^^10^

In this analysis of data from the largest prospective AF registry in South Asia, the Kerala Atrial Fibrillation (KERALA-AF) Registry, we performed hierarchical clustering to explore clinical phenotypic clusters of AF patients. Second, we analysed the associations between these clusters with clinical outcomes, their treatment strategies and the impact of adherence to integrated care management.

## Methods

### Study participants

KERALA-AF (trial registration details: CTRI/2017/10/010097) is an ongoing prospective multicentre cohort study of patients with AF in Kerala, India, and is the largest prospective AF registry in South Asia. The protocol and studies of this registry have been published.^11^^,^^12^ The 53 independent centres involved in KERALA-AF recruited 3401 patients with AF during 2016–2017.

### Inclusion and exclusion criteria

In this analysis, we included all patients in the registry, excluding those who did not complete one-year follow-up.

### Study outcomes

The outcomes of this study included all-cause mortality, major adverse cardiovascular events (MACE), and composite bleeding events. MACE were defined as the composite of cardiovascular disease (CVD) mortality, cerebrovascular accident (CVA), transient ischaemic attack (TIA), systemic embolism (SE), or hospitalisation for acute coronary syndrome (ACS), heart failure (HF) or arrhythmia. The composite bleeding outcome included gastrointestinal bleeding, intracranial haemorrhage, and minor bleeding.

### Definitions of ABC pathway

We assessed adherence to holistic or integrated care management based on the ABC pathway^13^ according to “A”, “B”, and “C” criteria based on the patient's baseline features. AF patients meeting all criteria were considered adherent to the ABC pathway:

♦ “A” criterion: patient with high thromboembolic risk (male: CHA_2_DS_2_-VASc ≥1; female: CHA_2_DS_2_-VASc ≥2) who received oral anticoagulants (OAC) was considered as adherent. Patients with low thromboembolic risk (male: CHA_2_DS_2_-VASc 0; female: CHA_2_DS_2_-VASc 1) who did not receive OAC were also regarded as adherent.
♦ “B” criterion: Because KERALA-AF was not designed to assess European Heart Rhythm Association (EHRA) scores of AF at the time of its original design, actual symptom severity scores were unavailable. AF-related symptoms were recorded in KERALA-AF, therefore, to quantify symptom control in AF patients, we considered patients with numbers of AF-related symptoms of ≤2 as adherent to the B criterion.
♦ “C” criterion: We considered the other most common comorbidities in patients with AF: HF, hypertension, dyslipidemia, coronary artery disease (CAD), and prior CVA. This criterion requires the above conditions to receive guideline-defined optimal drug treatments, which were defined as follows: (i) HF: receiving ACE inhibitors (ACEI) or angiotensin receptor blockers (ARB) and beta-blockers; (ii) hypertension: patients were considered adherent if their systolic blood pressure <140 mmHg and diastolic blood pressure <90 mmHg; if patients did not have records of blood pressure, they were also considered adherent if receiving beta-blockers or ACEI/ARB or calcium channel blockers (CCB) or diuretics. Although the KERALA-AF registry collected whether the patient had diabetes mellitus (DM) or not, it did not specifically collect information on the use of antidiabetic drugs; (iii) dyslipidemia: receiving statins; (iv) CAD: receiving ACEI or ARB or beta-blockers, and statins; (v) prior CVA or TIA: receiving statins. Patients with all of the above conditions receiving optimal treatment were considered adherent to the ABC pathway criteria.

### Extracted covariates

We collected demographic characteristics (e.g., age, sex), history of diseases (e.g., prior CVA, prior TIA), comorbidities (e.g., HF, hypertension), medications (e.g., warfarin, aspirin), AF treatment strategies (e.g., catheter ablation, pacemaker implantation), and imaging features (e.g., left ventricular ejection fraction [LVEF], left atrium [LA] size).

### Ethical approval and informed consent

The KERALA-AF registry study followed the principles in the Declaration of Helsinki and received ethical approvals from several ethics committees (Institutional Ethics Committee, Ananthapuri Hospitals and Research Institute; Institutional Ethics Committee, Sree Chitra Tirunal Institute of Medical Sciences and Technology; Ethics Committee, Lisie Heart Institute; Institutional Ethics Committee, Amrita Institute of Medical Sciences; Human Ethics Committee, Government Medical College, Trivandrum; Institutional Ethics Committee, Carithas Hospital, Kottayam; Institutional Ethics Committee, Sree Narayana Institute of Medical Sciences, Institutional Ethics Committee, Government Medical College, Calicut as well as by the Independent Ethics Committee of CSI–K). Since the data for this analysis were anonymised, no additional local ethical approval and informed consent was required.

### Hierarchical clustering approach

For this analysis, we employed Ward's method to identify different clusters. Initially, each sample is considered a separate cluster, and the squared Euclidean distance is used as a distance measurement between different clusters. We chose the combination of Ward's method and squared Euclidean distance which are validated in prior studies as being particularly effective for binary and categorical datasets due to its ability to minimise within-cluster variance.^6^^,^^14^ The distance metric between clusters is iteratively updated during the aggregation process, with greater distances indicating larger differences among clusters. Ultimately, the number of clusters selected for analysis was determined by observing the merging distance of different clusters shown in the dendrogram.

### Sensitivity analysis

To further verify the reliability of hierarchical clustering, we performed a sensitivity analysis using K-means clustering. In K-means clustering, the optimal number of clusters in K-means is determined using the “elbow method”. By plotting the explained variance against the number of clusters, we identified the inflection point where further addition of clusters resulted in insignificant model improvement. We then compared the phenotype clusters from K-means with those from hierarchical clustering to assess the stability of the hierarchical clustering method. However, since hierarchical clustering is more suitable for datasets with categorical variables, we finally used the hierarchical clustering results for subsequent analysis.

### Statistical analysis

For the hierarchical clustering analysis, we used 44 baseline features collected from the KERALA-AF registry with no more than 25% missing values (shown in Supplementary Table S1; maximum missing value proportion was 20.6%), including demographics, AF-related parameters, comorbidities, and imaging features. The threshold of 25% was selected to balance the goals of maintaining data integrity and retaining as many important variables as possible for meaningful analysis. We applied multivariate imputation by chained equations (MICE) to address missing data and ensure the robustness of the final analysis.^15^ This approach has been employed in previous clinical studies to impute missing values with different proportions. For example, Overtchouk et al. included missing data with the highest proportion of 24% in their analysis of the *French Transcatheter Aortic Valve Implantation* registry.^16^ Similarly, Moore et al. used MICE to impute 44% of the missing variables in their cohort.^17^ Additionally, Mishra et al. compared the performance of MICE for missing data with three proportions: 20%, 30% and 50%, the mean square error after MICE was similar for the missing proportions of 20% and 30% (both less than 0.1).^18^ This suggested that MICE worked reliably to fill in missing data when the proportion was less than 30%. Therefore, we employed MICE with random forests (with 25 imputations) to handle missing data, utilising the “miceforest” package in Python.

The results of the analysis of variance showed the nonnormal distribution of continuous variables in this study, therefore, they were expressed as median with interquartile range and compared among groups by using the Kruskal–Wallis test. Categorical variables were expressed as numbers with percentages, and differences among groups were assessed using Fisher's exact test. Some transformed categorical variables are defined in Supplementary Table S2. Then, we used multivariate logistic regression model analyses to determine differences in the risk of clinical outcomes across clusters, using the cluster with the lowest risk for each outcome event as the reference group, and the results were presented as odds ratios (OR) and 95% confidence intervals (CI). Covariates used for adjusting the logistic regression models included beta-blockers, rate-limiting CCB, digoxin, ACEI, ARB, dihydropyridine CCB, diuretics, statins, Class I antiarrhythmic drugs (AAD), Class III AAD, antiplatelet agents, anticoagulants, pacemaker implantation, surgery for AF, cardiac defibrillator implantation, left atrial appendage occlusion, and catheter ablation.

For the ABC pathway for AF, we assessed adherence to the A criterion, B criterion, C criterion, and ABC criteria in the whole cohort and each cluster separately. Subsequently, multivariate logistic regression models were used to evaluate the effect of ABC criteria adherence (vs. non-ABC adherence) and the impact of meeting 1–3 ABC criteria (vs. 0 ABC criteria) on all-cause mortality and MACE, adjusted for age, sex, type of AF, diabetes mellitus, HF, hypertension, dyslipidaemia, CAD, and prior CVA/TIA.

Hierarchical clustering was performed in Python (version 3.11.4, Python Software Foundation, Beaverton, OR, USA). Logistic regression analysis was performed in R (version 4.2.1, R Foundation for Statistical Computing, Vienna, Austria). Other comparisons were performed in SPSS (version 29.0.0.1, IBM Corporation, Armonk, NY, USA). Two-tailed *P* < 0.05 was considered as statistically significant.

### Role of funding sources

The Kerala-AF registry was supported by the Kerala Chapter of Cardiological Society of India through a one-time research grant No. CSI/IEC/2017. The funder did not play a role in the design or running of the study nor the analysis of results. No funding was received towards the analysis and writing of this manuscript.

## Results

After excluding 73 patients with incomplete follow-up, 3348 AF patients were included in this analysis. Median (IQR) age was 65.0 (56.0–74.0) years; 1634 (48.8%) were male; and median CHA_2_DS_2_-VASc was 3.0 (2.0–4.0).

**Fig. 1:**
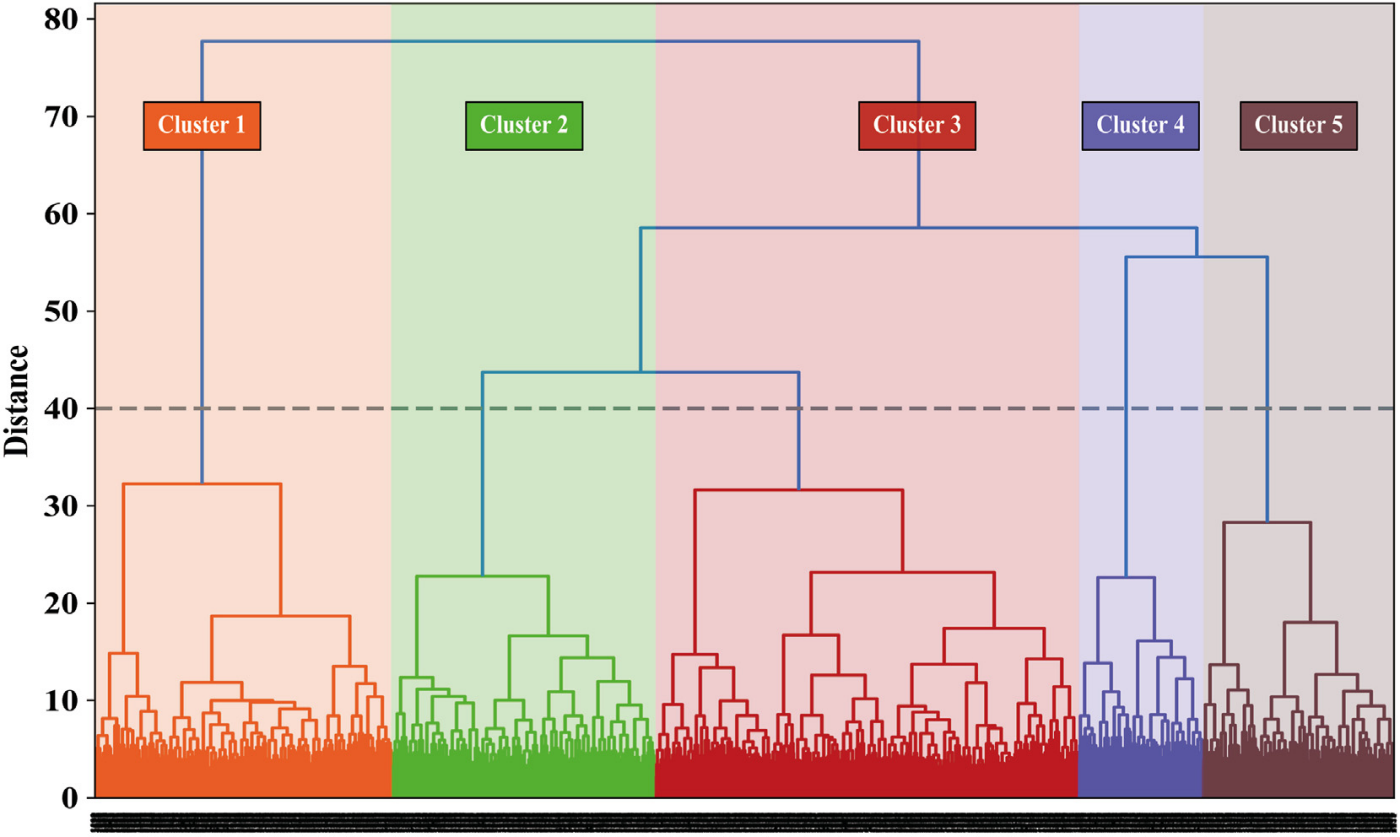
**Clustering process of this study.** The cluster analysis strategy was to minimise the variance of attribute differences within clusters using Ward's approach and the algorithm of squared Euclidean distance. Initially each sample is considered as a separate cluster, the distance metric between clusters is repeatedly updated during the aggregation of clusters, and finally formed different clusters with similar characteristics. The complete hierarchical clustering process was visualised as a dendrogram, where different vertical lines indicated different clusters and the Y-axis represented the distance measure of different clusters with the further away from the end of the tree and the greater the differences between clusters. By observing the dendrogram generated by the clustering process, the median Y-axis value was around 40, and the distance between the last four mergers in the clustering process was significantly greater than the previous mergers, therefore 5 clusters were selected.

**Fig. 2:**
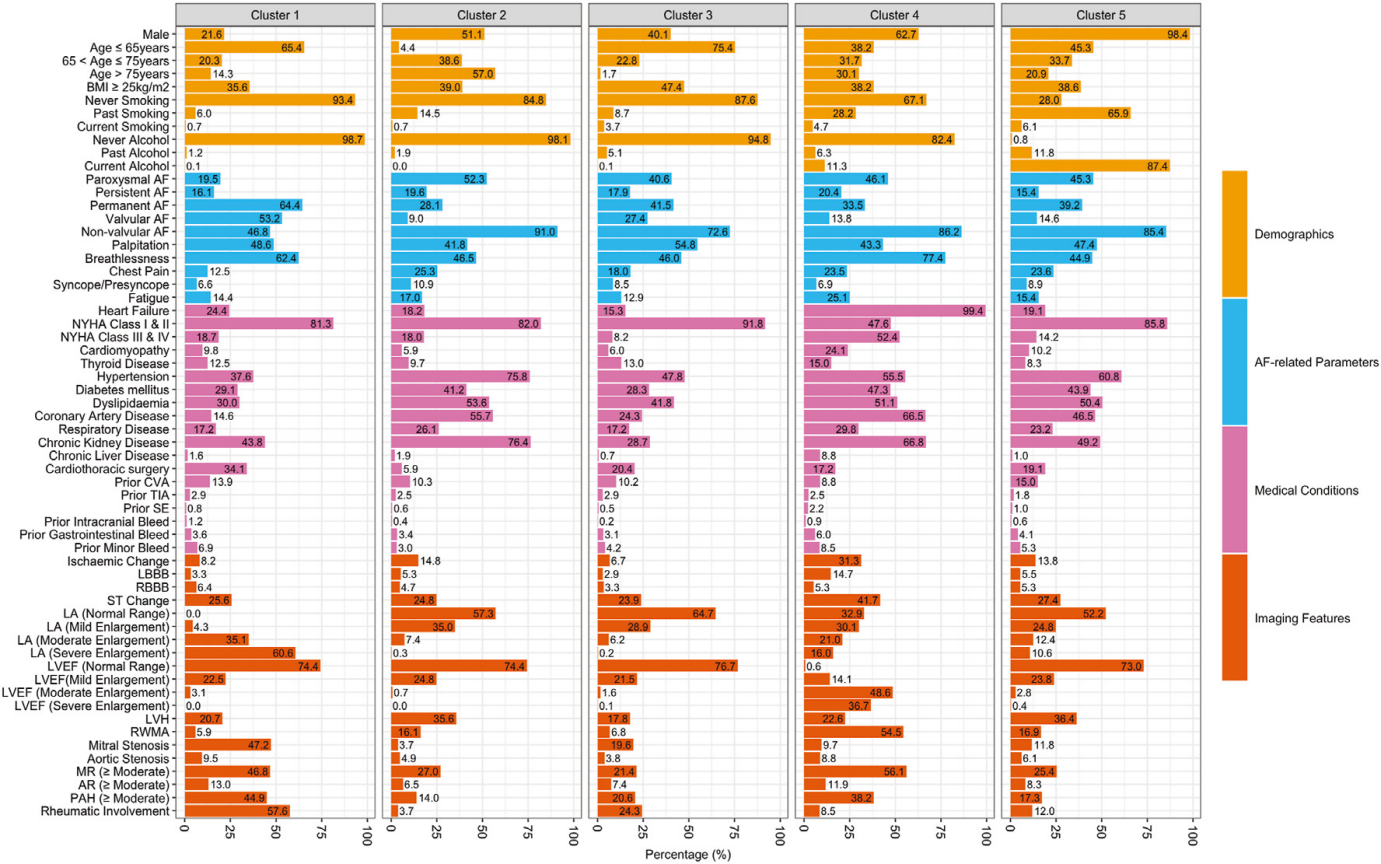
**Patient characteristics of different clusters by hierarchical clustering.** AF, atrial fibrillation; AR, aortic regurgitation; BMI, body mass index; CVA, cerebrovascular accident; LBBB, left bundle branch block; LVH, left ventricular hypertrophy; MR, mitral regurgitation; NYHA, New York Heart Association; PAH, pulmonary hypertension; RBBB, right bundle branch block; RWMA, regional wall motion abnormalities; SE, systemic embolism; TIA, transient ischaemic attack.

By observing the dendrogram generated after hierarchical clustering (Fig. 1), the median value of the Y-axis was approximately 40, and the distance between the last four mergers in the clustering process was significantly greater than the previous mergers, thus showing a clear ‘jump’. Therefore, the following five clusters were identified in this study (baseline characteristics are shown in Fig. 2 and Supplementary Table S3).

### Cluster 1: patients aged ≤65 years with rheumatic conditions

769 (23.0%) patients were included in Cluster 1. The median (IQR) age was 60.0 (51.0–70.0) years, with 503 patients (65.4%) being ≤65 years.

Compared with the other clusters, Cluster 1 had the highest proportion of valvular AF (53.2%), previous cardiothoracic surgery (34.1%), moderate-to-severe enlargement of LA (95.7%), and rheumatic involvement (57.6%), as well as the highest proportion of valvular anomalies among all clusters (*P* < 0.001 for each condition).

Patients in Cluster 1 were more likely to have permanent AF than those in the other clusters. The proportions of patients with high thromboembolic risk and high bleeding risk in Cluster 1 were 71.0% and 26.3%, respectively (*P* < 0.001 for each condition).

### Cluster 2: patients aged >65 years with multi-comorbidities

677 (20.2%) patients were included in Cluster 2. The median (IQR) age was 77.0 (71.0–82.0) years, with 647 patients (95.6%) >65 years. In comparison with other clusters, Cluster 2 had the highest proportion of hypertension (75.8%), dyslipidaemia (53.6%), and chronic kidney disease [CKD] (76.4%) [*P* < 0.001 for each condition], pointing to the presence of cardiovascular-kidney-metabolic (CKM) syndrome.^19^

Patients in Cluster 2 were more likely to have paroxysmal AF than those in the other clusters. Additionally, the proportions of patients with high thromboembolic risk and high bleeding risk in Cluster 2 were 99.9% and 80.5%, respectively (*P* < 0.001 for each condition).

### Cluster 3: patients aged ≤65 years with fewer comorbidities

There were 1091 (32.6%) patients in Cluster 3. The median (IQR) age was 60.0 (52.0–65.0) years, with 823 patients (75.4%) ≤65 years. Cluster 3 had the lowest proportions of HF, DM, respiratory disease, and CKD compared with the other clusters (*P* < 0.001 for each condition). The proportions of patients with high thromboembolic risk and high bleeding risk in Cluster 3 were 78.0% and 20.3%, respectively (*P* < 0.001 for each condition).

### Cluster 4: heart failure patients with multi-comorbidities

Cluster 4 included 319 (9.5%) patients. The median (IQR) age was 69.0 (61.0–78.0) years, with 96 patients (30.1%) being age >75 years. HF was prevalent in 99.4% of patients and significantly higher than other clusters (*P* < 0.001). Cluster 4 had the highest proportion of cardiomyopathy, thyroid disease, DM, CAD and chronic liver disease (*P* < 0.001 for each condition). The proportions of patients with high thromboembolic risk and high bleeding risk in Cluster 4 were 100.0% and 60.8%, respectively (*P* < 0.001 for each condition).

### Cluster 5: male patients with lifestyle-related risk factors

There were 492 (14.7%) patients in Cluster 5. The median (IQR) age was 67.0 (59.0–74.0) years, and 484 (98.4%) were males. Compared to the other clusters, Cluster 5 had the highest proportion of past/present smokers at 72.0% and past/present alcohol drinkers at 99.2% (*P* < 0.001 for each condition). The proportion of prior CVA in Cluster 5 was also the highest. The proportions of patients with high thromboembolic risk and high bleeding risk in Cluster 5 were 89.0% and 70.9%, respectively (*P* < 0.001 for each condition).

### Sensitivity analysis by K-means clustering

Using K-means clustering, the elbow method determined an optimal k of 5 (Supplementary Figure S1), so the same five clusters were identified with hierarchical clustering (baseline characteristics are shown in Fig. 2 and Supplementary Table S4). Cluster_k-means_ 1 (n = 802): *Patients aged >65 years with multiple comorbidities*, suggesting CKM (>65 years: 100.0%, hypertension: 76.7%, dyslipidaemia: 55.9%, CKD: 75.6%), corresponding to Cluster 2 of hierarchical cluster analysis. Cluster_k-means_ 2 (n = 916): *Patients aged ≤65 years with fewer comorbidities (*≤65 years: 85.7%, proportions of HF, DM, respiratory disease, and CKD compared with others, corresponding to Cluster 3 of hierarchical cluster analysis. Cluster_k-means_ 3 (n = 792): *patients aged ≤65 years with rheumatic conditions* (≤65 years: 73.6%, valvular AF: 53.2%, previous cardiothoracic surgery: 38.5%, moderate-to-severe enlargement of LA: 98.0%, rheumatic involvement: 57.6%), which corresponds to Cluster 1 of hierarchical cluster analysis. Cluster_k-means_ 4 (n = 483): *male patients with lifestyle-related risk factors* (males: 98.6%, past/present smokers: 75.2%, past/present alcohol drinkers at 100.0%), corresponding to Cluster 5 of the hierarchical cluster. Cluster_k-means_ 5 (n = 355): *HF patients with multiple comorbidities* (HF: 98.3%, cardiomyopathy: 23.1%, thyroid disease: 14.9%, DM: 48.7%, CAD: 68.5%), which corresponds to Cluster 4 of hierarchical cluster. The K-means clustering results closely mirrored the hierarchical clustering, identifying the same five distinct clinical phenotypes in the patient cohort, further validating the robustness of the clustering analysis.

### Therapeutic management of clusters

**Fig. 3:**
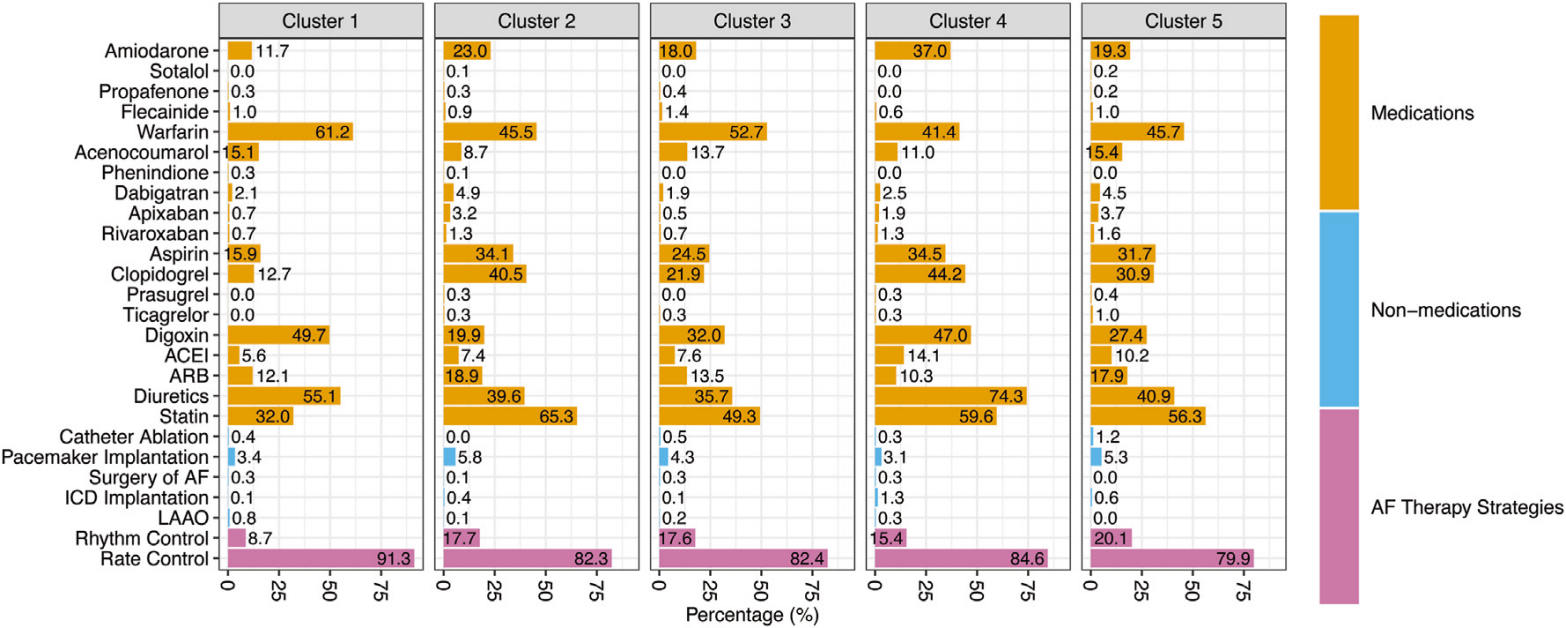
**AF treatment managment of different clusters by hierarchical clustering.** ACEI, angiotensin-converting enzyme inhibitors; AF, atrial fibrillation; ARB, angiotensin II receptor blockers; CCB, calcium channel blockers; ICD, Implantable cardioverter defibrillator; LAAO, left atrial appendage occlusion.

Fig. 3 and Supplemental Table S5 summarise the treatments of AF in the five clusters. Cluster 2 and Cluster 4 had relatively higher rates of antiplatelet agents use (57.6% and 57.7%, respectively; *P* < 0.001). Cluster 1 had the highest use of OAC, while Cluster 4 had the lowest use of OAC (*P* < 0.001). Among OAC, vitamin K antagonists were more frequently used in Cluster 1 (*P* < 0.001), whereas non-vitamin K antagonist OAC were used in <10.0% of all clusters (*P* < 0.001), likely due to the prevalence of valvular AF in this cluster. Combined use of OAC and antiplatelet therapy was more frequent in Clusters 2 and 5 but less common in Cluster 1 (*P* < 0.001). A rhythm control strategy was more commonly utilised in Cluster 5, while a rate control strategy was more common in Cluster 1 (*P* < 0.001).

### Adverse cardiovascular outcomes among clusters

During the one-year follow-up period, the cumulative incidence of all-cause mortality was 8.1% in Cluster 3, 10.4% in Cluster 5, 12.9% in Cluster 1, 17.7% in Cluster 2, and 21.3% in Cluster 4 (*P* < 0.001). The incidence of MACE was lowest in Cluster 3 (19.2%), while the cumulative incidence of MACE was 22.6%, 24.3%, 30.6%, and 32.6% in Clusters 5, 1, 2 and 4 respectively (*P* < 0.001) [Supplementary Figure S2].

**Fig. 4:**
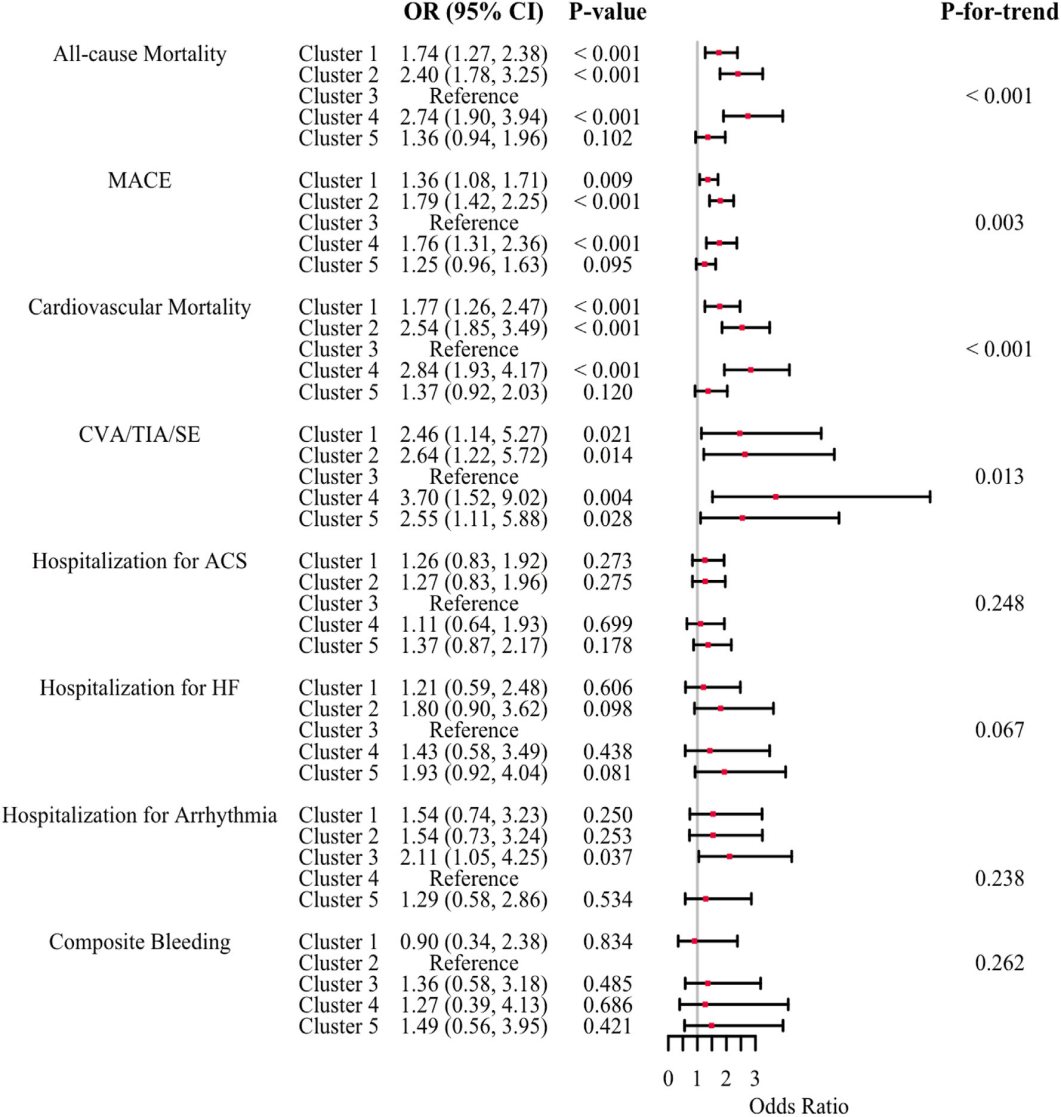
**Risk for outcomes on multivariate logistic regression analysis among different clusters by hierarchical clustering.** Adjust for beta-blockers, rate-limiting calcium channel blockers, digoxin, angiotensin-converting enzyme inhibitors, angiotensin II receptor blockers, dihydropyridine calcium channel blockers, diuretics, statins, Class I AAD, Class III AAD, antiplatelet agents, anticoagulants, pacemaker implantation, surgery for atrial fibrillation, cardiac defibrillator implantation, left atrial appendage occlusion, and catheter ablation. AAD, antiarrhythmic drug; ACS, acute coronary syndrome; CI, confidence interval; CVA, cerebrovascular accident; HF, heart failure; MACE, major adverse cardiovascular events; OR, odds ratio; SE, systemic embolism; TIA, transient ischemic attack.

Fig. 4 presents the results of the multivariate logistic regression analysis for various outcomes across clusters. Compared to Cluster 3, the risk of all-cause mortality was not significantly different for Cluster 5, whereas the other three clusters had higher risks of all-cause mortality (Cluster 1: OR 1.74, 95% CI 1.27–2.38, *P* < 0.001; Cluster 2: OR 2.40, 95% CI 1.78–3.25, *P* < 0.001; Cluster 4: OR 2.74, 95% CI 1.90–3.94, *P* < 0.001).

There was no significant difference in the risk of MACE in Cluster 5 compared to Cluster 3, whereas the risk of MACE was significantly higher in the other three clusters (Cluster 1: OR 1.36, 95% CI 1.08–1.71, *P* = 0.009; Cluster 2: OR 1.79, 95% CI 1.42–2.25, *P* < 0.001; Cluster 4: OR 1.76, 95% CI 1.31–2.36, *P* < 0.001).

For the individual endpoints within MACE, the CVA/TIA/SE risk was significantly higher for Clusters 1, 2, 4 and 5 compared with Cluster 3 (Fig. 4). The risks of ACS, HF and arrhythmia hospitalisation did not differ significantly between the clusters, except that Cluster 3 had a significantly higher risk of arrhythmic hospitalisation than Cluster 4 (OR 2.11, 95% CI 1.05–4.25, *P* = 0.037). There were no significant differences in the risk of composite bleeding events among the clusters.

### ABC pathway adherence

**Fig. 5:**
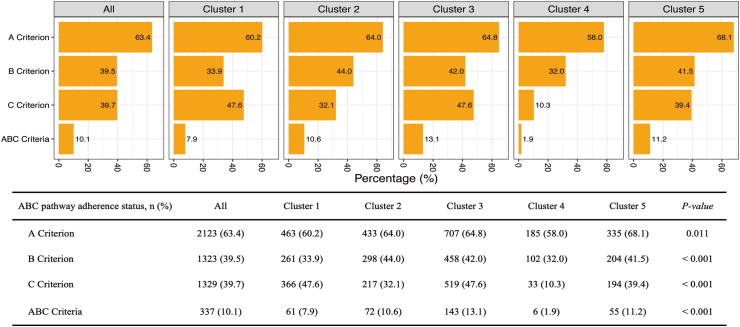
**The A criterion, B criterion, C criterion, and ABC criteria in the whole cohort and each cluster by hierarchical clustering**.

Fig. 5 illustrates adherence of A criterion, B criterion, C criterion, and full ABC pathway adherence in the overall cohort and different clusters. Adherence to the A criterion for the overall cohort was approximately 60%, generally similar across different clusters. Adherence to the B and C criteria in the overall cohort was about 40%, significantly different across clusters. Both were lowest in Cluster 4 (B criterion: 32.0%; C criterion: 10.3%). The proportion of ABC pathway adherence was about 1 in 10, with Cluster 1 and Cluster 4 having less than 10% adherence.

**Fig. 6:**
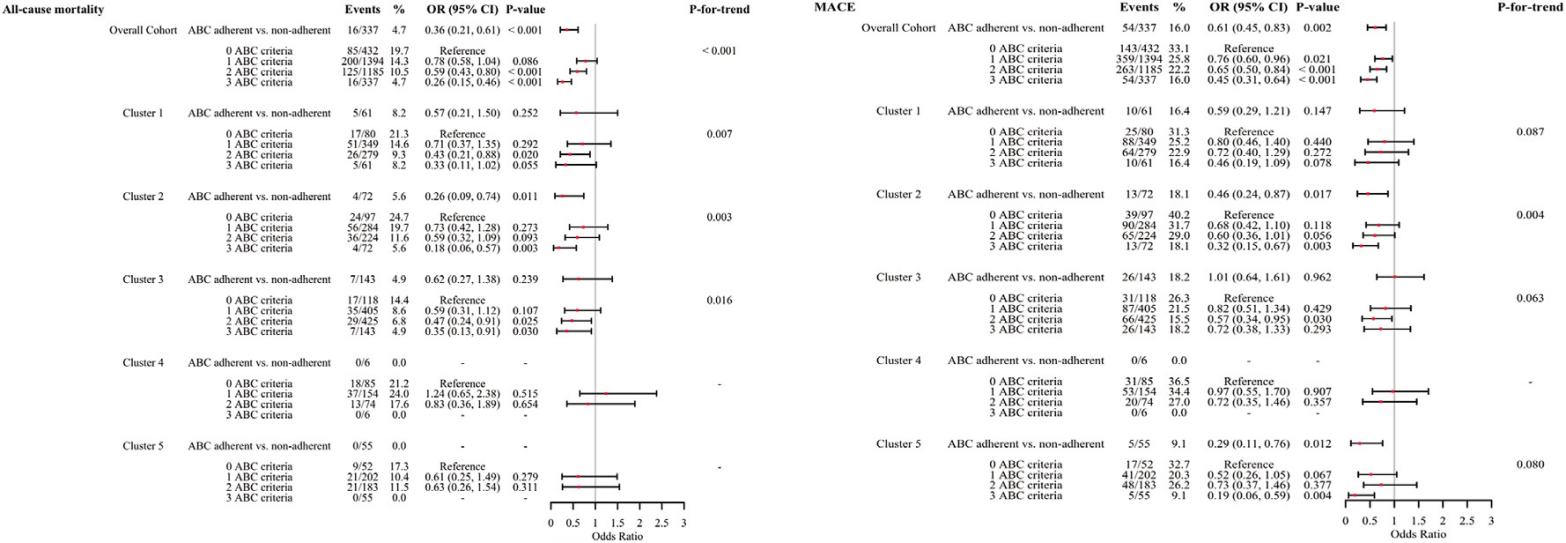
**Impact of adherence to the ABC pathway on all-cause mortality and mace in the whole cohort and each cluster by hierarchical clustering.** Adjusted for age, sex, type of atrial fibrillation, diabetes mellitus, heart failure, hypertension, dyslipidaemia, coronary artery disease, and prior cerebrovascular disease or transient ischaemic attack. CI, confidence interval; MACE, major adverse cardiovascular events; OR, odds ratio.

Multivariate logistic regression analyses (Fig. 6) indicated that in the overall cohort, compared with no integrated care, fully ABC pathway adherence was associated with reduced all-cause mortality (OR 0.26, 95% CI 0.15–0.46, *P* < 0.001) and MACE (OR 0.45, 95% CI 0.31–0.46, *P* < 0.001). Similar trends were evident in different clusters, although the limited sample size of some clusters resulted in statistically insignificant *P* values.

Fig. 6 demonstrates a graded effect of ABC pathway adherence when comparing the impact of 0, 1, 2 and 3 criteria on mortality and MACE in the overall cohort and within each cluster.

## Discussion

In this cluster analysis from the KERALA-AF registry, five clinical phenotypes were identified in South Asian patients with AF. (*i*) Patients aged ≤65 years with rheumatic conditions. (*ii*) Patients aged >65 years with multiple comorbidities. (*iii*) Patients aged ≤65 years with few comorbidities. (*iv*) HF patients with multiple comorbidities. (*v*) Male patients with lifestyle-related risk factors. Second, different prognostic outcomes were associated with different phenotypic clusters within our study. Patients aged >65 years with multi-comorbidity and HF patients with multi-comorbidity had a worse prognosis, with higher rates of all-cause mortality, MACE, cardiovascular mortality and CVA/TIA/SE. Third, ABC pathway adherence was low, but adherence to the ABC pathway significantly reduced the risk of all-cause mortality and MACE, even in the clusters.

### Importance of identifying disease-related clinical phenotypes

AF is rarely an isolated disease but is often a complication of cardiac and non-cardiac disease. Cluster analysis can categorise patients based on various clinical characteristics and potentially identify clinically relevant but subtle phenotypes.

Due to the differences in clinical phenotypes, the outcome varies significantly among different clusters. For example, patients in Cluster 1 were mainly patients aged ≤65 years with rheumatic conditions and significantly enlarged LA due to mitral stenosis. Rheumatic heart disease commonly results in mitral stenosis^20^; however, if treated with surgical valve replacement, the outcome is more favourable.^21^ Therefore, outcomes in Cluster 1 were relatively better than in Cluster 2 or Cluster 4, whose age tended to be more advanced, along with multiple comorbidities. These factors are well-known to be the determinants of adverse outcomes in patients with AF.^21^^,^^22^ In contrast, patients in Cluster 3 and Cluster 5 had fewer comorbidities and, perhaps unsurprisingly, had relatively better outcomes.

Cluster 2, although defined as a multi-comorbidities group, had the highest proportion of CKD and dyslipidaemia. These characteristics suggested that Cluster 2 might manifest CKM syndrome,^19^ a complex clinical syndrome involving multiple disorders of the cardiac, renal, and metabolic systems that interact with each other and contribute to the progression of the disease.^23^^,^^24^ This finding highlights the potential importance of identifying CKM in patients with AF, and future studies are necessary to clarify the impact of the presence of CKM on the prognosis of AF.

### Comparisons with prior clustering studies related to AF in other ethnicities

Several studies have used clustering to evaluate the clinical phenotypes and their associated outcomes in patients with AF. Vitolo et al. analysed with hierarchical clustering the AF patients in the AMADEUS and BOREALIS trials and identified four different clinical phenotypes with unique clinical characteristics and various outcomes, as follows: Cluster 1 with low rates of cardiovascular risk factors and comorbidities; Cluster 2 with high burden of cardiovascular risk factors; Cluster 3 with high burden of cardiovascular comorbidities; and Cluster 4 with the highest rates of non-cardiovascular comorbidities. When compared with Cluster 1, only Cluster 4 was associated with an increased risk of all-cause mortality and major bleeding.^25^

In a study by Ogawa and colleagues, AF patients were categorised into six comorbidity clusters, each with significantly distinct outcomes.^8^ Compared to Cluster 2, Cluster 1 (patients with a mean age of 48.3 years, characterised by a low prevalence of risk factors and comorbidities) was associated with a lower risk of adverse outcomes, whereas other Clusters 3–6 (patients with atherosclerotic risk factors and comorbidities) were linked to all-cause mortality and cardiovascular mortality.

Saito et al. utilised the K-prototype method for cluster analysis in the SAKURA AF registry and further externally validated this in the RAFFINE AF registry.^26^ Five clusters were identified with varying characteristics, and when compared with Cluster 1 (males with a mean age of 57.1 years and few comorbidities), other clusters were related to a higher risk of all-cause mortality and composite events (including major bleeding, stroke, myocardial infarction and all-cause mortality), with Cluster 4 (female patients and with prior heart failure) experiencing the worst outcomes.

Our findings are partially consistent with previous studies while presenting some unique characteristics. Similar to clusters identified by Vitolo et al.,^25^ Ogawa et al.,^8^ and Saito et al.,^26^ we also identified a low-risk cluster comprising patients aged ≤65 years with fewer comorbidities (Cluster 3 in our study). Similarly, patients aged >65 years with multiple comorbidities had a significantly worse prognosis, as shown in our Cluster 2 (patients aged >65 years with CKM) and Cluster 4 (HF patients with multiple comorbidities), which coincided with prior works. However, our study highlights differences between the comorbidity clusters, with Clusters 2 and 4 underscoring the importance of CKM and HF, respectively. This refined distinction between these two high-risk groups is unique to our study and has not been clearly defined in previous studies. Moreover, we also identified Cluster 1 (patients aged ≤65 years with rheumatic conditions) and Cluster 5 (male patients with lifestyle-related risk factors), which were not mentioned in prior studies but showed significant risks of adverse prognosis in our study, further emphasising the heterogeneity of AF patients in different regions and populations.

Although these studies all utilised clustering methods, the phenotypic characteristics of the clusters varied significantly among different studies, indicating that AF patients have significant clinical heterogeneity in other regions or ethnicities. Prior studies were based on analyses from clinical trials or registries conducted in developed countries,^8^^,^^25^^,^^26^ but data from the developing area were scarce. Our study demonstrates the utility of cluster analysis for AF patients in developing countries. Moreover, patients in the KERALA-AF Registry had their characteristics such as low anti-coagulation therapy, high use of rate control strategy and high comorbidities burden, making it a representative cohort for clinical phenotypes analysis with clustering.

In our study, the percentage of adherence to the ABC pathway in the KERALA-AF Registry was low, with only 10.1% of patients fully adherent. However, prior studies have shown significant differences in the ABC pathway adherence across regions and ethnicities. Krittayaphong et al. reported an ABC pathway adherence rate of 42.7% among AF patients in the COOL-AF Registry in Thailand and that patients who adhered to the ABC pathway (either fully or partially) had a lower risk of all-cause mortality and cardiovascular events compared to non-adherents.^27^ Similarly, in the Gloria-AF registry (mainly involved European and North American AF populations), Romiti et al. reported an ABC pathway adherence rate of 26.4% and that adherence to the ABC pathway significantly reduced the risks of all-cause mortality and adverse cardiovascular events. Compared with these regions, whether in Europe, North America or other Asian countries (e.g. Thailand), the adherence of the South Asian AF population in our study was significantly lower, which reflects the differences in medical services and resources.

The difference in adherence to the ABC pathway among the five clusters also showed significant differences. The proportion of patients fully adhering to the ABC pathway was relatively higher in Cluster 3 (13.1%) and lowest in Cluster 4 (1.9%). Among “ABC criteria”, the greatest difference among the five clusters was concerning adherence with the C criterion, especially in Cluster 4, whereby only 10.3% had adherence. Consistently, the outcomes in Cluster 4 were the worst among the five clusters. Similar to our study, Krittayaphong et al. conducted another clustering analysis of the COOL-AF Registry, which identified three clusters: Cluster 1 consisted of patients whose mean age was 75.6 years, characterised by multiple comorbidities; Cluster 2 included patients with a mean age of 56.5 years and few comorbidities; and Cluster 3 consisted of patients with an average age of 74.3 years and few comorbidities. They further reported that Clusters 1 and 3 benefited from ABC pathway adherence, whereas the effect was non-significant for Cluster 2.

Our findings also demonstrated that adherence to the ABC pathway was protective in some clusters. For example, adherence to the ABC pathway in Cluster 2 was associated with a lower risk of all-cause mortality and MACE. However, due to the short follow-up period and extremely low adherence to the ABC pathway, our study failed to achieve satisfactory statistical power to clarify the effect of ABC pathway adherence on different clusters.

Nevertheless, our findings further emphasised the great heterogeneity in clinical characteristics and management of AF. Importantly, adherence to the ABC pathway was associated with improved clinical outcomes in patients with AF, as recommended by current guidelines.^10^

Cluster analysis identifies clinically relevant phenotypic groups of AF patients, based on which more specific management strategies can help improve outcomes. For example, patients in Cluster 1 are mainly with rheumatic conditions, and anticoagulant therapy is particularly important because these patients have an extremely high risk of thromboembolism. For patients in Cluster 3, the treatment of comorbidities should be intensified due to their high comorbidities burden and high risk of MACE. In contrast, lifestyle modifications could be a practical approach for patients in Cluster 5. Therefore, this clustering approach may help identify patient subgroups for implementing phenotype-specific treatments and optimise clinical decision-making. Future prospective studies and randomised controlled trials are required to validate the clinical utility of clustering and to determine whether specific treatments work better or worse within identified clusters.

Despite the overall low adherence in the South Asian AF population, ABC pathway adherence was still associated with a significant reduction in adverse outcomes. Moreover, the effect of ABC pathway adherence varied among different clusters. Considering our findings, more targeted interventions should be explored for improving ABC pathway adherence in different clinical phenotype clusters in the South Asian AF population, especially in high-risk clusters.

Besides, our cluster analysis revealed significant differences between different phenotype clusters, which provides a basis for constructing accurate personalised prediction tools. Predictive models for high-risk AF phenotypes can be constructed in the future based on essential variables such as electrocardiograms, echocardiographic parameters, and cardiac biomarkers. These tools are promising for improving the efficiency of healthcare resource allocation and the quality of clinical decision support. Additionally, cross-ethnic validation will further promote the universality of these cluster optimisation models in different populations, providing a basis for managing AF in other ethnic groups and regions.

There are several limitations to our analysis. First, this is a post hoc analysis from an observational registry with limited ability to analyse subgroups not pre-specified in the original study design. Some variables had much missing data so that reliability may be limited. Second, follow-up data from the KERALA-AF registry only recorded events during the follow-up period but without the specific date of occurrence, making it impossible to perform time-to-event analysis. Third, the clusters identified in this analysis were based on baseline characteristics only; however, risk is dynamic and may be altered by new complications or subsequent treatment decisions—i.e., patients may transition between clusters across follow-up. Fourth, our study recruited patients from hospitals, which may not apply to the general population. Fifth, although our selection of the optimal number of clusters for hierarchical clustering was based on scientific methodology and there were significant differences in characteristics and risks between clusters, the results of the cluster analyses should be interpreted with caution, as different algorithms or thresholds may result in different clusters being identified. Sixth, the limited number of patients who were fully adherent to the ABC pathway and the small number of events limited statistical power and some outcomes were not statistically significant or had huge 95% CIs. Also, our results for ABC adherence are only based on baseline data, and adherence rates may change subsequently. Lastly, although this study is derived from the largest South Asian AF registry, it is the first to identify clinical subtypes of South Asian AF with different risks and demonstrates the effectiveness of the ABC pathway in improving prognosis, future prospective trials with more rigorous designs are necessary to confirm our results.

Cluster analysis is an effective method for identifying patients with different clinical phenotypes in a given dataset, which has implications for clinical outcomes. Five major clinical groups were identified for the first time in South Asian AF patients: patients aged ≤65 years with rheumatic conditions, patients aged >65 years with multiple comorbidities, patients aged ≤65 years with few comorbidities, HF patients with multiple comorbidities, and male patients with lifestyle-related risk factors. Compared to patients aged ≤65 years with few comorbidities, other clusters were associated with a higher risk of major adverse outcomes. Based on this, clinicians can implement targeted interventions, improving outcomes and resource use. Despite poor ABC pathway adherence overall, such adherence still positively impacted clinical outcomes even in low-resource settings, with varying effects across clusters. Future research should focus on enhancing adherence and personalised care strategies for optimised management of these patient.

## Contributors

Jinbert Lordson Azariah (JLA), Narayanan Namboodiri (NN), Govindan Unni (GU), Jayagopal P B (JPB), and Bahuleyan Charantharayil Gopalan (BCG) contributed to the acquisition and interpretation of data. Yang Chen (YC), Bi Huang (BH), Ying Gue (YG), and Gregory Y.H. Lip (GYHL) initiated, planned, and designed the study. YC, BH, Yang Liu (YL), YG, and Garry McDowell (GM) conducted the literature review. YC and YG conducted the statistical analyses, as well as the preparation of figures and tables, with statistical validation performed by GM and GYHL. The first draft of the manuscript was written by YC and BH. Peter Calvert (PC), YL, YG, Dhiraj Gupta (DG), GU, JPB, GYHL, and BCG revised the manuscript. YG, GM, GYHL, and BCG ensured management of the project and the study. All named authors meet the criteria for authorship as outlined by the International Committee of Medical Journal Editors, take collective responsibility for the integrity of the work, and have provided their approval for its publication.

## Data sharing statement

Data may be made available upon reasonable request. The R code used are available from the corresponding author upon request.

## Declaration of interests

All authors report no conflicts of interest.
