# Supplementary material for: Phenotypes of South Asian patients with atrial fibrillation and holistic integrated care management: cluster analysis of data from KERALA-AF Registry
Source: Lancet Reg Health Southeast Asia. 2024 Nov 14;31:100507. doi: 10.1016/j.lansea.2024.100507 (PMC11609390; doi:10.1016/j.lansea.2024.100507)
Supplement: Supplementary Data [file mmc1.docx]

**Supplementary Table S1. Missing Values of Variables Included in Clustering Process.**

| **Varialbes** | **Percentage of missing, n (%)** |
| --- | --- |
| New York Heart Association Class | 4 (0.1) |
| Age | 11 (3.6) |
| Height | 206 (6.2) |
| Weight | 117 (3.5) |
| Echocardiography Ischaemic Change | 287 (8.6) |
| Echocardiography Left Bundle Branch Block | 261 (7.8) |
| Echocardiography Right Bundle Branch Block | 261 (7.8) |
| Echocardiography ST Change | 290 (8.7) |
| Left atrium diameter | 691 (20.6) |
| Left Ventricular Ejection Fraction | 508 (15.2) |
| Left Ventricular Hypertrophy | 334 (10.0) |
| Regional Wall Motion Abnormalities | 314 (9.4) |
| Mitral Stenosis | 314 (9.4) |
| Aortic Stenosis | 325 (9.7) |
| Mitral Regurgitation (≥ Moderate) | 306 (9.1) |
| Aortic Regurgitation (≥ Moderate) | 322 (9.6) |
| Pulmonary Hypertension (≥ Moderate) | 308 (9.2) |
| Rheumatic Involvement | 323 (9.6) |

**Supplementary Table S2. Names and Definitions of Transformed Categorical Variables.**

| **Variables** | **Definition** |
| --- | --- |
| LA Enlargement |  |
| Normal Range | Male: LA diameter < 41mm  Female: LA diameter < 39mm |
| Mild | Male: 41mm ≤ LA diameter < 47mm  Female: 39mm ≤ LA diameter < 43mm |
| Moderate | Male: 47mm ≤ LA diameter < 52mm  Female: 43mm ≤ LA diameter < 47mm |
| Severe | Male: LA diameter ≥ 52mm  Female: LA diameter ≥ 47mm |
| LVEF |  |
| Normal Range | Male: LVEF > 51%  Female: LVEF > 53% |
| Mild | Male: 40% < LVEF ≤ 51%  Female: 40% < LVEF ≤ 53% |
| Moderate | 30% ≤ LVEF ≤ 40% |
| Severe | LVEF < 30% |
| Minor Bleeding | Any sign or symptom of haemorrhage (e.g., more bleeding than would be expected for a clinical circumstance, including bleeding found by imaging alone) that does not fit the criteria for major bleeding but does meet at least one of the following criteria.  a. requiring medical intervention by a healthcare professional.  b. leading to hospitalization or increased level of care.  c. prompting a doctor’s consultation. |
| Pulmonary Arterial Hypertension (≥ Moderate) | Right ventricular systolic pressure ≥ 50mmHg |
| High Bleeding Risk | HAS-BLED ≥ 3 |

Abbreviations: LA, left atrium; LVEF, left ventricular ejection fraction.

**Supplementary Table S3. Patient Baseline Characteristics of Different Clusters By Hierarchical Clustering.**

|  | **Cluster 1**  **(N = 769)** | **Cluster 2**  **(N = 677)** | **Cluster 3**  **(N = 1091)** | **Cluster 4**  **(N = 319)** | **Cluster 5**  **(N = 492)** | ***P-value*** |
| --- | --- | --- | --- | --- | --- | --- |
| Demographics |  |  |  |  |  |  |
| Male, n (%) | 166 (21.6) | 346 (51.1) | 438 (40.1) | 200 (62.7) | 484 (98.4) | < 0.001 |
| Age, years | 60.0 (51.0, 70.0) | 77.0 (71.0, 82.0) | 60.0 (52.0, 65.0) | 69.0 (61.0, 78.0) | 67.0 (59.0, 74.0) | < 0.001 |
| Category Age, n (%) |  |  |  |  |  | < 0.001 |
| Age ≤ 65years | 503 (65.4) | 30 (4.4) | 823 (75.4) | 122 (38.2) | 223 (45.3) |  |
| 65 < Age ≤ 75years | 156 (20.3) | 261 (38.6) | 249 (22.8) | 101 (31.7) | 166 (33.7) |  |
| Age > 75years | 110 (14.3) | 386 (57.0) | 19 (1.7) | 96 (30.1) | 103 (20.9) |  |
| BMI, kg/m^2^ | 23.6 (21.1, 26.3) | 24.1 (21.6, 26.4) | 24.8 (22.3, 27.2) | 24.1 (21.2, 26.4) | 24.0 (22.0, 26.2) | < 0.001 |
| BMI ≥ 25kg/m^2^, n (%) | 274 (35.6) | 264 (39.0) | 517 (47.4) | 122 (38.2) | 190 (38.6) | < 0.001 |
| Smoking, n (%) |  |  |  |  |  | < 0.001 |
| Never | 718 (93.4) | 574 (84.8) | 956 (87.6) | 214 (67.1) | 138 (28.0) |  |
| Past | 46 (6.0) | 98 (14.5) | 95 (8.7) | 90 (28.2) | 324 (65.9) |  |
| Current | 5 (0.7) | 5 (0.7) | 40 (3.7) | 15 (4.7) | 30 (6.1) |  |
| Alcohol, n (%) |  |  |  |  |  | < 0.001 |
| Never | 759 (98.7) | 664 (98.1) | 1034 (94.8) | 263 (82.4) | 4 (0.8) |  |
| Past | 9 (1.2) | 13 (1.9) | 56 (5.1) | 20 (6.3) | 58 (11.8) |  |
| Current | 1 (0.1) | 0 (0.0) | 1 (0.1) | 36 (11.3) | 430 (87.4) |  |
| AF Classification, n (%) |  |  |  |  |  | < 0.001 |
| Paroxysmal AF | 150 (19.5) | 354 (52.3) | 443 (40.6) | 147 (46.1) | 223 (45.3) |  |
| Persistent AF | 124 (16.1) | 133 (19.6) | 195 (17.9) | 65 (20.4) | 76 (15.4) |  |
| Permanent AF | 495 (64.4) | 190 (28.1) | 453 (41.5) | 107 (33.5) | 193 (39.2) |  |
| Type of AF, n (%) |  |  |  |  |  | < 0.001 |
| Valvular AF | 409 (53.2) | 61 (9.0) | 299 (27.4) | 44 (13.8) | 72 (14.6) |  |
| Non-valvular AF | 360 (46.8) | 616 (91.0) | 792 (72.6) | 275 (86.2) | 420 (85.4) |  |
| AF Symptom, n (%) |  |  |  |  |  |  |
| Palpitation | 374 (48.6) | 283 (41.8) | 598 (54.8) | 138 (43.3) | 233 (47.4) | < 0.001 |
| Breathlessness | 480 (62.4) | 315 (46.5) | 502 (46.0) | 247 (77.4) | 221 (44.9) | < 0.001 |
| Chest Pain | 96 (12.5) | 171 (25.3) | 196 (18.0) | 75 (23.5) | 116 (23.6) | < 0.001 |
| Syncope/Presyncope | 51 (6.6) | 74 (10.9) | 93 (8.5) | 22 (6.9) | 44 (8.9) | 0.044 |
| Fatigue | 111 (14.4) | 115 (17.0) | 141 (12.9) | 80 (25.1) | 76 (15.4) | < 0.001 |
| Medical Condition, n (%) |  |  |  |  |  |  |
| Heart Failure | 188 (24.4) | 123 (18.2) | 167 (15.3) | 317 (99.4) | 94 (19.1) | < 0.001 |
| NYHA Class |  |  |  |  |  | < 0.001 |
| Class I & II | 625 (81.3) | 555 (82.0) | 1002 (91.8) | 152 (47.6) | 422 (85.8) |  |
| Class III & IV | 144 (18.7) | 122 (18.0) | 89 (8.2) | 167 (52.4) | 70 (14.2) |  |
| Cardiomyopathy | 75 (9.8) | 40 (5.9) | 66 (6.0) | 77 (24.1) | 50 (10.2) | < 0.001 |
| Thyroid Disease | 96 (12.5) | 66 (9.7) | 142 (13.0) | 48 (15.0) | 41 (8.3) | 0.009 |
| Hypertension | 289 (37.6) | 513 (75.8) | 522 (47.8) | 177 (55.5) | 299 (60.8) | < 0.001 |
| Diabetes mellitus | 224 (29.1) | 279 (41.2) | 309 (28.3) | 151 (47.3) | 216 (43.9) | < 0.001 |
| Dyslipidaemia | 231 (30.0) | 363 (53.6) | 456 (41.8) | 163 (51.1) | 248 (50.4) | < 0.001 |
| Coronary Artery Disease | 112 (14.6) | 377 (55.7) | 265 (24.3) | 212 (66.5) | 229 (46.5) | < 0.001 |
| Respiratory Disease | 132 (17.2) | 177 (26.1) | 188 (17.2) | 95 (29.8) | 114 (23.2) | < 0.001 |
| Chronic Kidney Disease | 337 (43.8) | 517 (76.4) | 313 (28.7) | 213 (66.8) | 242 (49.2) | < 0.001 |
| Chronic Liver Disease | 12 (1.6) | 13 (1.9) | 8 (0.7) | 28 (8.8) | 5 (1.0) | < 0.001 |
| Cardiothoracic surgery | 262 (34.1) | 40 (5.9) | 223 (20.4) | 55 (17.2) | 94 (19.1) | < 0.001 |
| Prior CVA | 107 (13.9) | 70 (10.3) | 111 (10.2) | 28 (8.8) | 74 (15.0) | 0.004 |
| Prior TIA | 22 (2.9) | 17 (2.5) | 32 (2.9) | 8 (2.5) | 9 (1.8) | 0.765 |
| Prior SE | 6 (0.8) | 4 (0.6) | 5 (0.5) | 7 (2.2) | 5 (1.0) | 0.040 |
| Prior Intracranial Bleed | 9 (1.2) | 3 (0.4) | 2 (0.2) | 3 (0.9) | 3 (0.6) | 0.081 |
| Prior Gastrointestinal Bleed | 28 (3.6) | 23 (3.4) | 34 (3.1) | 19 (6.0) | 20 (4.1) | 0.202 |
| Prior Minor Bleed | 53 (6.9) | 20 (3.0) | 46 (4.2) | 27 (8.5) | 26 (5.3) | < 0.001 |
| Electrocardiography Information, n (%) | | | | | | |
| Ischaemic Change | 63 (8.2) | 100 (14.8) | 73 (6.7) | 100 (31.3) | 68 (13.8) | < 0.001 |
| LBBB | 25 (3.3) | 36 (5.3) | 32 (2.9) | 47 (14.7) | 27 (5.5) | < 0.001 |
| RBBB | 49 (6.4) | 32 (4.7) | 36 (3.3) | 17 (5.3) | 26 (5.3) | 0.040 |
| ST Change | 197 (25.6) | 168 (24.8) | 261 (23.9) | 133 (41.7) | 135 (27.4) | < 0.001 |
| Echocardiography Information, n (%) | | | | | | |
| Left Atrium Enlargement |  |  |  |  |  | < 0.001 |
| Normal Range | 0 (0.0) | 388 (57.3) | 706 (64.7) | 105 (32.9) | 257 (52.2) |  |
| Mild | 33 (4.3) | 237 (35.0) | 315 (28.9) | 96 (30.1) | 122 (24.8) |  |
| Moderate | 270 (35.1) | 50 (7.4) | 68 (6.2) | 67 (21.0) | 61 (12.4) |  |
| Severe | 466 (60.6) | 2 (0.3) | 2 (0.2) | 51 (16.0) | 52 (10.6) |  |
| Left Ventricular Ejection Fraction | | | | | | < 0.001 |
| Normal Range | 572 (74.4) | 504 (74.4) | 837 (76.7) | 2 (0.6) | 359 (73.0) |  |
| Mild | 173 (22.5) | 168 (24.8) | 235 (21.5) | 45 (14.1) | 117 (23.8) |  |
| Moderate | 24 (3.1) | 5 (0.7) | 18 (1.6) | 155 (48.6) | 14 (2.8) |  |
| Severe | 0 (0.0) | 0 (0.0) | 1 (0.1) | 117 (36.7) | 2 (0.4) |  |
| LVH | 159 (20.7) | 241 (35.6) | 194 (17.8) | 72 (22.6) | 179 (36.4) | < 0.001 |
| RWMA | 45 (5.9) | 109 (16.1) | 74 (6.8) | 174 (54.5) | 83 (16.9) | < 0.001 |
| Mitral Stenosis | 363 (47.2) | 25 (3.7) | 214 (19.6) | 31 (9.7) | 58 (11.8) | < 0.001 |
| Aortic Stenosis | 73 (9.5) | 33 (4.9) | 41 (3.8) | 28 (8.8) | 30 (6.1) | < 0.001 |
| MR (≥ Moderate) | 360 (46.8) | 183 (27.0) | 234 (21.4) | 179 (56.1) | 125 (25.4) | < 0.001 |
| AR (≥ Moderate) | 100 (13.0) | 44 (6.5) | 81 (7.4) | 38 (11.9) | 41 (8.3) | < 0.001 |
| PAH (≥ Moderate) | 345 (44.9) | 95 (14.0) | 225 (20.6) | 122 (38.2) | 85 (17.3) | < 0.001 |
| Rheumatic Involvement | 443 (57.6) | 25 (3.7) | 265 (24.3) | 27 (8.5) | 59 (12.0) | < 0.001 |

Abbreviations: AF, atrial fibrillation; AR, aortic regurgitation; BMI, body mass index; CVA, cerebrovascular accident; LBBB, left bundle branch block; LVH, left ventricular hypertrophy; MR, mitral regurgitation; NYHA, New York Heart Association; PAH, pulmonary arterial hypertension; RBBB, right bundle branch block; RWMA, regional wall motion abnormalities; SE, systemic embolism; TIA, transient ischaemic attack.

**Supplementary Table S4. Patient Baseline Characteristics of Different Clusters By K-means Clustering for Sensitivity Analysis.**

|  | **Cluster_k-means_ 1**  **(N = 802)** | **Cluster_k-means_ 2**  **(N = 916)** | **Cluster_k-means_ 3**  **(N = 792)** | **Cluster_k-means_ 4**  **(N = 483)** | **Cluster_k-means_ 5**  **(N = 355)** | ***P-value*** |
| --- | --- | --- | --- | --- | --- | --- |
| Demographics |  |  |  |  |  |  |
| Male, n (%) | 353 (44.0) | 382 (41.7) | 202 (25.5) | 476 (98.6) | 221 (62.3) | < 0.001 |
| Category Age, n (%) |  |  |  |  |  | < 0.001 |
| Age ≤ 65years | 0 (0) | 785 (85.7) | 583 (73.6) | 205 (42.4) | 128 (36.1) |  |
| 65 < Age ≤ 75years | 353 (44.0) | 131 (14.3) | 160 (20.2) | 175 (36.2) | 114 (32.1) |  |
| Age > 75years | 449 (56.0) | 0 (0) | 49 (6.2) | 103 (21.3) | 113 (31.8) |  |
| BMI ≥ 25kg/m^2^, n (%) | 310 (38.7) | 437 (47.7) | 287 (36.2) | 197 (40.8) | 219 (38.3) | < 0.001 |
| Smoking, n (%) |  |  |  |  |  | < 0.001 |
| Never | 696 (86.8) | 819 (89.4) | 719 (90.8) | 120 (24.8) | 246 (69.3) |  |
| Past | 98 (12.2) | 81 (8.8) | 66 (8.3) | 313 (64.8) | 95 (26.8) |  |
| Current | 8 (1.0) | 16 (1.7) | 7 (0.9) | 50 (10.4) | 14 (3.9) |  |
| Alcohol, n (%) |  |  |  |  |  | < 0.001 |
| Never | 794 (99.0) | 885 (96.6) | 743 (93.8) | 0 (0) | 302 (85.1) |  |
| Past | 8 (1.0) | 31 (3.4) | 14 (1.8) | 79 (16.4) | 24 (6.8) |  |
| Current | 0 (0) | 0 (0) | 35 (4.4) | 404 (83.6) | 29 (8.2) |  |
| AF Classification, n (%) |  |  |  |  |  | < 0.001 |
| Paroxysmal AF | 435 (54.2) | 383 (41.8) | 129 (16.3) | 221 (45.8) | 149 (42.0) |  |
| Persistent AF | 245 (30.5) | 362 (39.5) | 524 (66.2) | 177 (36.6) | 130 (36.6) |  |
| Permanent AF | 122 (15.2) | 171 (18.7) | 139 (17.6) | 85 (17.6) | 76 (21.4) |  |
| Type of AF, n (%) |  |  |  |  |  | < 0.001 |
| Valvular AF | 77 (9.6) | 259 (28.3) | 444 (56.1) | 57 (11.8) | 48 (13.5) |  |
| Non-valvular AF | 725 (90.4) | 657 (71.7) | 348 (43.9) | 426 (88.2) | 307 (86.5) |  |
| AF Symptom, n (%) |  |  |  |  |  |  |
| Palpitation | 331 (41.3) | 513 (56.0) | 406 (51.3) | 218 (45.1) | 158 (44.5) | < 0.001 |
| Breathlessness | 365 (45.5) | 396 (43.2) | 519 (65.5) | 212 (43.9) | 273 (76.9) | < 0.001 |
| Chest Pain | 204 (25.4) | 161 (17.6) | 93 (11.7) | 115 (23.8) | 81 (22.8) | < 0.001 |
| Syncope/Presyncope | 92 (11.5) | 79 (8.6) | 47 (5.9) | 45 (9.3) | 21 (5.9) | 0.044 |
| Fatigue | 131 (16.3) | 122 (13.3) | 112 (14.1) | 76 (15.7) | 82 (23.1) | < 0.001 |
| Medical Condition, n (%) |  |  |  |  |  |  |
| Heart Failure | 115 (14.3) | 125 (13.6) | 210 (26.5) | 90 (18.6) | 349 (98.3) | < 0.001 |
| NYHA Class |  |  |  |  |  | < 0.001 |
| Class I & II | 674 (84.0) | 861 (94.0) | 635 (80.2) | 421 (87.2) | 165 (46.5) |  |
| Class III & IV | 128 (16.0) | 55 (6.0) | 157 (19.8) | 62 (12.8) | 190 (53.5) |  |
| Cardiomyopathy | 50 (6.2) | 48 (5.2) | 91 (11.5) | 37 (7.7) | 82 (23.1) | < 0.001 |
| Thyroid Disease | 92 (11.5) | 118 (12.9) | 94 (11.9) | 36 (7.5) | 53 (14.9) | 0.010 |
| Hypertension | 615 (76.7) | 422 (46.1) | 249 (31.4) | 313 (64.8) | 201 (56.6) | < 0.001 |
| Diabetes mellitus | 333 (41.5) | 251 (27.4) | 200 (25.3) | 222 (46.0) | 173 (48.7) | < 0.001 |
| Dyslipidaemia | 448 (55.9) | 359 (39.2) | 210 (26.5) | 256 (53.0) | 188 (53.0) | < 0.001 |
| Coronary Artery Disease | 417 (52.0) | 203 (22.2) | 95 (12.0) | 237 (49.1) | 243 (68.5) | < 0.001 |
| Respiratory Disease | 210 (26.2) | 133 (14.5) | 147 (18.6) | 108 (22.4) | 108 (30.4) | < 0.001 |
| Chronic Kidney Disease | 606 (75.6) | 229 (25.0) | 316 (39.9) | 232 (48.0) | 239 (67.3) | < 0.001 |
| Chronic Liver Disease | 14 (1.7) | 4 (0.4) | 12 (1.5) | 6 (1.2) | 30 (0.9) | < 0.001 |
| Cardiothoracic surgery | 45 (5.6) | 181 (19.8) | 305 (38.5) | 78 (16.1) | 65 (18.3) | < 0.001 |
| Prior CVA | 90 (11.2) | 98 (10.7) | 94 (11.9) | 67 (13.9) | 41 (11.5) | 0.508 |
| Prior TIA | 24 (3.0) | 25 (2.7) | 20 (2.5) | 10 (2.1) | 9 (2.5) | 0.897 |
| Prior SE | 3 (0.4) | 5 (0.5) | 6 (0.8) | 5 (1.0) | 8 (2.3) | 0.015 |
| Prior Intracranial Bleed | 4 (0.5) | 2 (0.2) | 9 (0.3) | 2 (0.1) | 3 (0.8) | 0.143 |
| Prior Gastrointestinal Bleed | 32 (4.0) | 21 (2.3) | 30 (3.8) | 19 (3.9) | 22 (6.2) | 0.021 |
| Prior Minor Bleed | 34 (4.2) | 32 (3.5) | 49 (6.2) | 26 (5.4) | 31 (8.7) | 0.001 |
| Electrocardiography Information, n (%) | | | | | | |
| Ischaemic Change | 105 (13.1) | 59 (6.4) | 61 (7.7) | 67 (13.9) | 112 (31.5) | < 0.001 |
| LBBB | 37 (4.6) | 22 (2.4) | 25 (3.2) | 24 (5.0) | 59 (16.6) | < 0.001 |
| RBBB | 41 (5.1) | 25 (2.7) | 49 (6.2) | 27 (5.6) | 18 (5.1) | 0.012 |
| ST Change | 228 (28.4) | 191 (20.9) | 203 (25.6) | 135 (28.0) | 137 (38.6) | < 0.001 |
| Echocardiography Information, n (%) | | | | | | |
| Left Atrium Enlargement | |  |  |  |  | < 0.001 |
| Normal Range | 435 (54.2) | 650 (71.0) | 0 (0) | 275 (56.9) | 96 (27.0) |  |
| Mild | 267 (33.3) | 263 (28.7) | 16 (2.0) | 140 (29.0) | 117 (33.0) |  |
| Moderate | 98 (12.2) | 3 (0.3) | 280 (35.4) | 53 (11.0) | 82 (23.1) |  |
| Severe | 2 (0.2) | 0 (0) | 496 (62.6) | 15 (3.1) | 60 (16.9) |  |
| Left Ventricular Ejection Fraction | | | | | | < 0.001 |
| Normal Range | 614 (76.6) | 725 (79.1) | 582 (73.5) | 353 (73.1) | 0 (0) |  |
| Mild | 188 (23.4) | 185 (20.2) | 187 (23.6) | 111 (23.0) | 67 (18.9) |  |
| Moderate | 0 (0) | 6 (0.7) | 22 (2.8) | 19 (3.9) | 169 (47.6) |  |
| Severe | 0 (0) | 0 (0) | 1 (0.1) | 0 (0) | 119 (33.5) |  |
| LVH | 281 (35.0) | 154 (16.8) | 135 (16.0) | 180 (37.3) | 95 (26.8) | < 0.001 |
| RWMA | 106 (13.2) | 56 (6.1) | 41 (5.2) | 79 (16.4) | 203 (57.2) | < 0.001 |
| Mitral Stenosis | 27 (3.4) | 194 (21.2) | 400 (50.5) | 37 (7.7) | 33 (9.3) | < 0.001 |
| Aortic Stenosis | 47 (5.9) | 29 (3.2) | 76 (9.6) | 26 (5.4) | 27 (7.6) | < 0.001 |
| MR (≥ Moderate) | 196 (24.4) | 195 (21.3) | 359 (45.3) | 111 (10.3) | 220 (62.0) | < 0.001 |
| AR (≥ Moderate) | 59 (7.4) | 61 (6.7) | 110 (13.9) | 33 (6.8) | 41 (11.5) | < 0.001 |
| PAH (≥ Moderate) | 126 (15.7) | 160 (17.5) | 377 (47.6) | 64 (13.3) | 145 (40.8) | < 0.001 |
| Rheumatic Involvement | 22 (2.7) | 235 (25.7) | 497 (62.8) | 33 (6.8) | 32 (9.0) | < 0.001 |

Abbreviations: AF, atrial fibrillation; AR, aortic regurgitation; BMI, body mass index; CVA, cerebrovascular accident; LBBB, left bundle branch block; LVH, left ventricular hypertrophy; MR, mitral regurgitation; NYHA, New York Heart Association; PAH, pulmonary arterial hypertension; RBBB, right bundle branch block; RWMA, regional wall motion abnormalities; SE, systemic embolism; TIA, transient ischaemic attack.

**Supplementary Table S5. Baseline AF Treatment Managment of Different Clusters By Hierarchical Clustering.**

|  | **Cluster 1**  **(N = 769)** | **Cluster 2**  **(N = 677)** | **Cluster 3**  **(N = 1091)** | **Cluster 4**  **(N = 319)** | **Cluster 5**  **(N = 492)** | ***P-value*** |
| --- | --- | --- | --- | --- | --- | --- |
| Medications, n (%) |  |  |  |  |  |  |
| Amiodarone | 90 (11.7) | 156 (23.0) | 196 (18.0) | 118 (37.0) | 95 (19.3) | < 0.001 |
| Sotalol | 0 (0.0) | 1 (0.1) | 0 (0.0) | 0 (0.0) | 1 (0.2) | 0.423 |
| Propafenone | 2 (0.3) | 2 (0.3) | 4 (0.4) | 0 (0.0) | 1 (0.2) | 0.853 |
| Flecainide | 8 (1.0) | 6 (0.9) | 15 (1.4) | 2 (0.6) | 5 (1.0) | 0.777 |
| Warfarin | 471 (61.2) | 308 (45.5) | 575 (52.7) | 132 (41.4) | 225 (45.7) | < 0.001 |
| Acenocoumarol | 116 (15.1) | 59 (8.7) | 149 (13.7) | 35 (11.0) | 76 (15.4) | 0.001 |
| Phenindione | 2 (0.3) | 1 (0.1) | 0 (0.0) | 0 (0.0) | 0 (0.0) | 0.348 |
| Dabigatran | 16 (2.1) | 33 (4.9) | 21 (1.9) | 8 (2.5) | 22 (4.5) | 0.001 |
| Apixaban | 5 (0.7) | 22 (3.2) | 5 (0.5) | 6 (1.9) | 18 (3.7) | < 0.001 |
| Rivaroxaban | 5 (0.7) | 9 (1.3) | 8 (0.7) | 4 (1.3) | 8 (1.6) | 0.336 |
| Aspirin | 122 (15.9) | 231 (34.1) | 267 (24.5) | 110 (34.5) | 156 (31.7) | < 0.001 |
| Clopidogrel | 98 (12.7) | 274 (40.5) | 239 (21.9) | 141 (44.2) | 152 (30.9) | < 0.001 |
| Prasugrel | 0 (0.0) | 2 (0.3) | 0 (0.0) | 1 (0.3) | 2 (0.4) | 0.164 |
| Ticagrelor | 0 (0.0) | 2 (0.3) | 3 (0.3) | 1 (0.3) | 5 (1.0) | 0.045 |
| Digoxin | 382 (49.7) | 135 (19.9) | 349 (32.0) | 150 (47.0) | 135 (27.4) | < 0.001 |
| ACEI | 43 (5.6) | 50 (7.4) | 83 (7.6) | 45 (14.1) | 50 (10.2) | < 0.001 |
| ARB | 93 (12.1) | 128 (18.9) | 147 (13.5) | 33 (10.3) | 88 (17.9) | < 0.001 |
| Diuretics | 424 (55.1) | 268 (39.6) | 390 (35.7) | 237 (74.3) | 201 (40.9) | < 0.001 |
| Statin | 246 (32.0) | 442 (65.3) | 538 (49.3) | 190 (59.6) | 277 (56.3) | < 0.001 |
| Classified Medications, n (%) | | | | | | |
| Class I AAD | 10 (1.3) | 8 (1.2) | 19 (1.7) | 2 (0.6) | 6 (1.2) | 0.602 |
| Beta Blockers | 409 (53.2) | 368 (54.4) | 626 (57.4) | 194 (60.8) | 277 (56.3) | 0.132 |
| Class III AAD | 90 (11.7) | 157 (23.2) | 196 (18.0) | 118 (37.0) | 96 (19.5) | < 0.001 |
| Non-dihydropyridine CCB | 155 (20.2) | 166 (24.5) | 223 (20.4) | 45 (14.1) | 86 (17.5) | 0.002 |
| Dihydropyridine CCB | 72 (9.4) | 108 (16.0) | 108 (9.9) | 32 (10.0) | 74 (15.0) | < 0.001 |
| VKA | 586 (76.2) | 368 (54.4) | 723 (66.3) | 167 (52.4) | 301 (61.2) | < 0.001 |
| NOAC | 26 (3.4) | 64 (9.5) | 34 (3.1) | 18 (5.6) | 48 (9.8) | < 0.001 |
| Anticoagulant | 612 (79.6) | 432 (63.8) | 755 (69.2) | 185 (58.0) | 347 (70.5) | < 0.001 |
| Antiplatelet Agent | 198 (25.7) | 390 (57.6) | 412 (37.8) | 184 (57.7) | 259 (52.6) | < 0.001 |
| Non-medications, n (%) |  |  |  |  |  |  |
| Catheter Ablation | 3 (0.4) | 0 (0.0) | 6 (0.5) | 1 (0.3) | 6 (1.2) | 0.053 |
| Pacemaker Implantation | 26 (3.4) | 39 (5.8) | 47 (4.3) | 10 (3.1) | 26 (5.3) | 0.136 |
| Surgery of AF | 2 (0.3) | 1 (0.1) | 3 (0.3) | 1 (0.3) | 0 (0.0) | 0.801 |
| ICD Implantation | 1 (0.1) | 3 (0.4) | 1 (0.1) | 4 (1.3) | 3 (0.6) | 0.022 |
| LAAO | 6 (0.8) | 1 (0.1) | 2 (0.2) | 1 (0.3) | 0 (0.0) | 0.076 |
| OAC and Antiplatelet Agents | 158 (20.5) | 238 (35.2) | 272 (24.9) | 91 (28.5) | 182 (37.0) | < 0.001 |
| Rhythm Control | 67 (8.7) | 120 (17.7) | 192 (17.6) | 49 (15.4) | 99 (20.1) | < 0.001 |
| Rate Control | 702 (91.3) | 557 (82.3) | 899 (82.4) | 270 (84.6) | 393 (79.9) | < 0.001 |
| Risk Scores |  |  |  |  |  |  |
| CHA_2_DS_2_-VASc | 2.0 (1.0, 3.0) | 4.0 (3.0, 5.0) | 2.0 (1.0, 3.0) | 4.0 (3.0, 5.0) | 3.0 (2.0, 4.0) | < 0.001 |
| High thromboembolic risk, n (%) | 546 (71.0) | 676 (99.9) | 851 (78.0) | 319 (100.0) | 438 (89.0) | < 0.001 |
| HAS-BLED | 2.0 (1.0, 3.0) | 3.0 (3.0, 4.0) | 1.0 (1.0, 2.0) | 3.0 (2.0, 4.0) | 3.0 (2.0, 4.0) | < 0.001 |
| High Bleeding Risk, n (%) | 202 (26.3) | 545 (80.5) | 221 (20.3) | 125 (60.8) | 349 (70.9) | < 0.001 |

Abbreviations: AAD, antiarrhythmic drug; ACEI, angiotensin-converting enzyme inhibitors; AF, atrial fibrillation; ARB, angiotensin II receptor blockers; CCB, calcium channel blocker; ICD, Implantable cardioverter defibrillator; LAAO, left atrial appendage occlusion; NOAC, non-vitamin K antagonist oral anticoagulants; VKA, vitamin K antagonist.


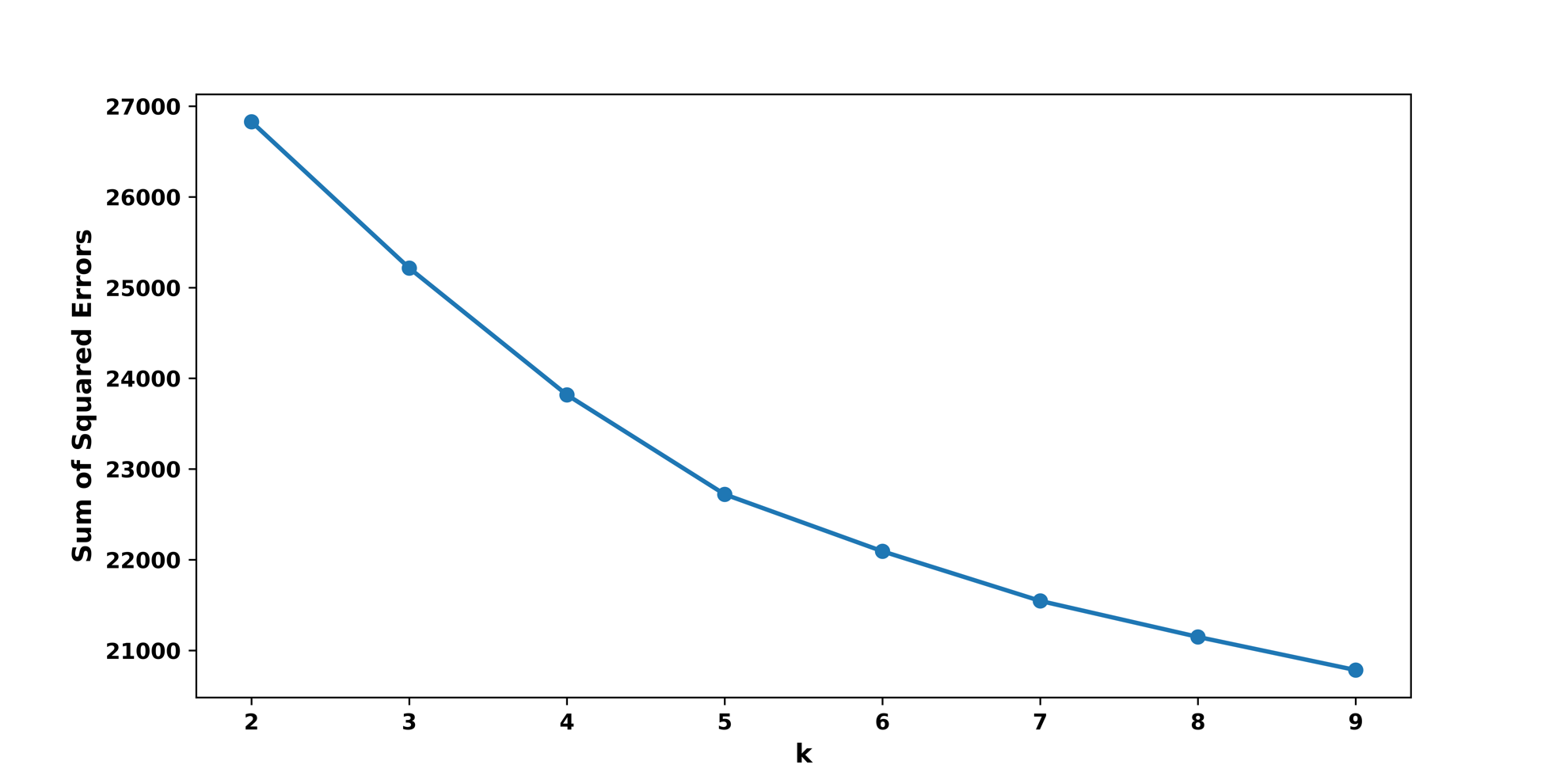


**Supplementary Figure S1. Optimal Number of Clusters (k) Determined by Elbow Method for K-means Clustering.** The graph displays the sum of squared errors plotted against different values of k (number of clusters). The optimal number of clusters is identified at k = 5, where the curve starts to flatten, indicating that increasing k beyond this point does not significantly reduce the sum of squared errors. This point is known as the “elbow,” where adding more clusters would lead to diminishing returns in terms of variance reduction.


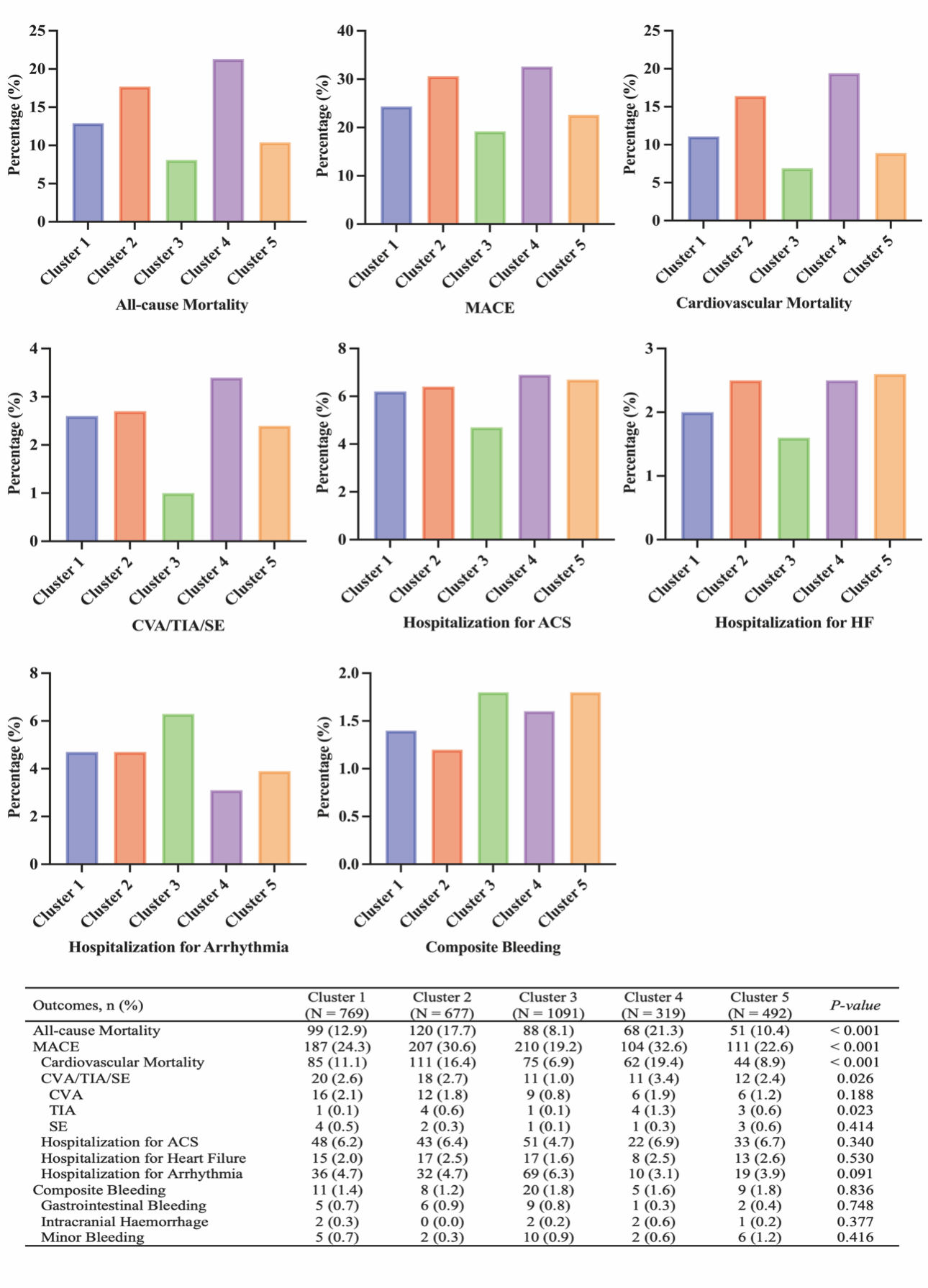


**Supplementary Figure S2. Outcomes of Different Clusters By Hierarchical Clustering During One-year Follow-up.** ACS, acute coronary syndrome; CVA, cerebrovascular accident; HF, heart failure; MACE, major adverse cardiovascular events; SE, systemic embolism; TIA, transient ischemic attack.
